# Evaluation of Safety and Efficacy of Prehospital Paramedic Administration of Sub-Dissociative Dose of Ketamine in the Treatment of Trauma-Related Pain in Adult Civilian Population

**DOI:** 10.7759/cureus.9567

**Published:** 2020-08-05

**Authors:** Alex Jabourian, Fanglong Dong, Kevin Mackey, Reza Vaezazizi, Troy W Pennington, Michael Neeki

**Affiliations:** 1 Emergency Medicine, Arrowhead Regional Medical Center, Colton, USA; 2 Emergency Medicine, Sacramento Regional Fire, Sacramento, USA; 3 Emergency Medicine, Inland Counties Emergency Medical Service Agency, San Bernardino, USA; 4 Emergency Medicine, Riverside County Emergency Medical Services Agency, Riverside, USA

**Keywords:** ketamine, civilian, adult, prehospital

## Abstract

Opiates are addicting and have a high potential for dependency. In the past decades, opiates remained the first-line pharmaceutical option of prehospital treatment for acute traumatic pain in the civilian population. Ketamine is an N-methyl-d-aspartate (NMDA) receptor antagonist that has analgesic properties and may serve as an alternative agent for the treatment of acute traumatic pain in prehospital settings. This study aims to assess the safety and efficacy of ketamine administration by paramedics in civilian prehospital settings for the treatment of acute traumatic pain.

This was a prospective observational study in San Bernardino, Riverside and Stanislaus counties. Patients were included if they were > 15 years of age with complaints of traumatic or burn-related pain. Patients were excluded if they received opiates up to six hours prior to or concurrently with ketamine administration. The dose administered was 0.3 mg/kg intravenously over five minutes with a maximum dose of 30 mg. The option to administer a second dose was available to paramedics if the patient continued to have pain after 15 minutes following the first administration. Paired-T tests were conducted to assess the change in the primary outcome (pain score) and secondary outcomes (e.g. systolic blood pressure, pulse, and respiratory rate). P-value<0.05 was considered to be statistically significant.

A total of 368 patients were included in the final analysis. The average age was 52.9 ± 23.1 years, and the average weight was 80.4 ± 22.2 kg. There was a statistically significant reduction in the pain score (9.13 ± 1.28 vs 3.7 ± 3.4, delta=5.43 ± 3.38, p<0.0001). Additionally, there was a statistically significant change in systolic blood pressure (143.42 ± 27.01 vs 145.65 ± 26.26, delta=2.22 ± 21.1, p=0.044), pulse (88.06 ± 18 vs 84.64 ± 15.92, delta= -3.42 ± 12.12, p<0.0001), and respiratory rate (19.04 ± 3.59 vs 17.74 ± 3.06, delta=-1.3 ± 2.96, p<0.0001).

The current study suggested that paramedics are capable of safely identifying the appropriate patients for the administration of sub-dissociative doses of ketamine in the prehospital setting. Furthermore, the current study suggested that ketamine may be an effective analgesic in a select group of adult trauma patients.

## Introduction

The medicinal effects of opium have been exploited for centuries, long before the synthesis of the first opiate drug. However, the extent of its addictive properties were not evident until the last several decades. Currently, 11.4 million people misuse prescription opiates in the United States, with an estimated 130 people dying every day from opioid-related drug overdoses [1]. In 2017, more than 47,600 deaths were attributed to opioid overdose. The cost of this epidemic in 2013 estimated almost $78.5 billion [2]. The economic burden of opiate addiction and misuse is projected to increase as opiate prescriptions are still readily distributed, with an estimated 58.5 per 100 Americans receiving an opiate prescription in 2017 [3]. Despite this public health emergency, opiates remain a first-line pharmaceutical option for patients with acute pain. In the prehospital setting, opiates - morphine and fentanyl - remain the recommended means of treatment for moderate to severe pain [4]. Alternative means of treating acute traumatic pain in a prehospital setting have been proposed, including the use of non-steroidal anti-inflammatory drugs. However, no unequivocal data existed to support a clinically significant reduction in pain with use of nonsteroidal anti-inflammatory drugs (NSAIDs) in the prehospital setting [5]. 

Ketamine may be a non-opioid alternative used for pain management. In 1982, Lodge et al. conclusively demonstrated that ketamine acts as an N-methyl-d-aspartate (NMDA) receptor antagonist [6]. However, further research showed that ketamine may also have opiate-receptor specific binding capacity, thus eliciting an analgesic effect [7]. Ketamine’s efficacy as an analgesic was first described in 1971 [8]. Since then, several studies have demonstrated the efficacy of ketamine as an analgesic in the hospital setting [9-12]. The vast majority of data available for pre-hospital administration of ketamine reflects on its utilization in military and combat environments [13-18].

To date, there were limited data on the use of ketamine in the civilian prehospital environment as an analgesic. This study aimed to evaluate the safety and efficacy of paramedic administration of ketamine as a prehospital analgesic in traumatically injured civilian patients. We hypothesize that the administration of ketamine in the prehospital setting by paramedics is safe and effective in reducing pain.

## Materials and methods

A prospective observational study was conducted by paramedics representing 30 advanced life support (ALS) transport providers in three California counties (San Bernardino, Riverside and Stanislaus). Prior authorization was obtained from Local Emergency Medical Services Agencies. Additionally, the protocol was approved by the Institutional Review Board at Arrowhead Regional Medical Center (Colton, CA). An oversight committee in each participating county regularly reviewed the data and addressed any discrepancies during the study period. The paramedics participating in the study were trained by their respective participating agencies regarding the ketamine administration inclusion and exclusion criteria and appropriate documentation. Patients were included in the study if they: were at least 15 years of age; had experienced an acute traumatic or burn injury; had a Glasgow Coma Scale (GCS) score of 15; would normally receive analgesia during routine care and/or transport, and had a pain score of at least “5” on a scale of 0 - 10, with 0 meaning “no pain” and 10 meaning “extremely severe pain”. The exclusion criteria were: GCS 14 or less; known or suspected pregnancy; known allergy to ketamine; known or suspected alcohol or drug intoxication; having received narcotic analgesia in any form within six hours of planned ketamine administration; and pain score less than "5" prior to the first dose of ketamine. Paramedics assessed each patient for the source of the pain to determine whether the pain was somatic or visceral. Paramedics were taught that visceral pain or chronic pain is less likely to respond to ketamine, and they should choose a different analgesic in those circumstances. However, if the patient met the inclusion criteria, then ketamine was preferred. 

Pain was assessed on a visual analogue pain scale ranging from 0 (no pain) to 10 (most severe pain). Pain assessment was performed by a paramedic prior to administration of ketamine. Reassessment of pain was performed every five minutes following administration. The pain scale measured at 15 minutes was used as the final pain scale. EMS personnel were instructed to document age, weight, chief complaint, initial vital signs (including systolic blood pressure), Glasgow coma scale, as well as past medical history, allergies and home medications used by the patient.

Paramedics were instructed to administer 0.3 mg/kg (max dose 30 mg) of ketamine as slow intravenous push over five minutes. An additional dose of 0.3 mg/kg (max dose 30 mg) was permitted if pain severity remained greater than five on the pain scale, 15 minutes following first administration. The time of ketamine administration and total dose were documented in the electronic patient care report, as well as any observed adverse effects.

All statistical analyses were conducted using the SAS software for Windows version 9.3 (Cary, North Carolina, USA). Descriptive statistics were presented as means and standard deviations for continuous variables, along with frequencies and proportions for categorical variables. Change in the pain scale and vital signs were analyzed using the paired T-test. Change in the pain scale was also compared between those who received one vs two doses of ketamine using independent T-test. All statistical analyses were two-sided. P-value<0.05 was considered to be statistically significant.

## Results

**Figure 1:**
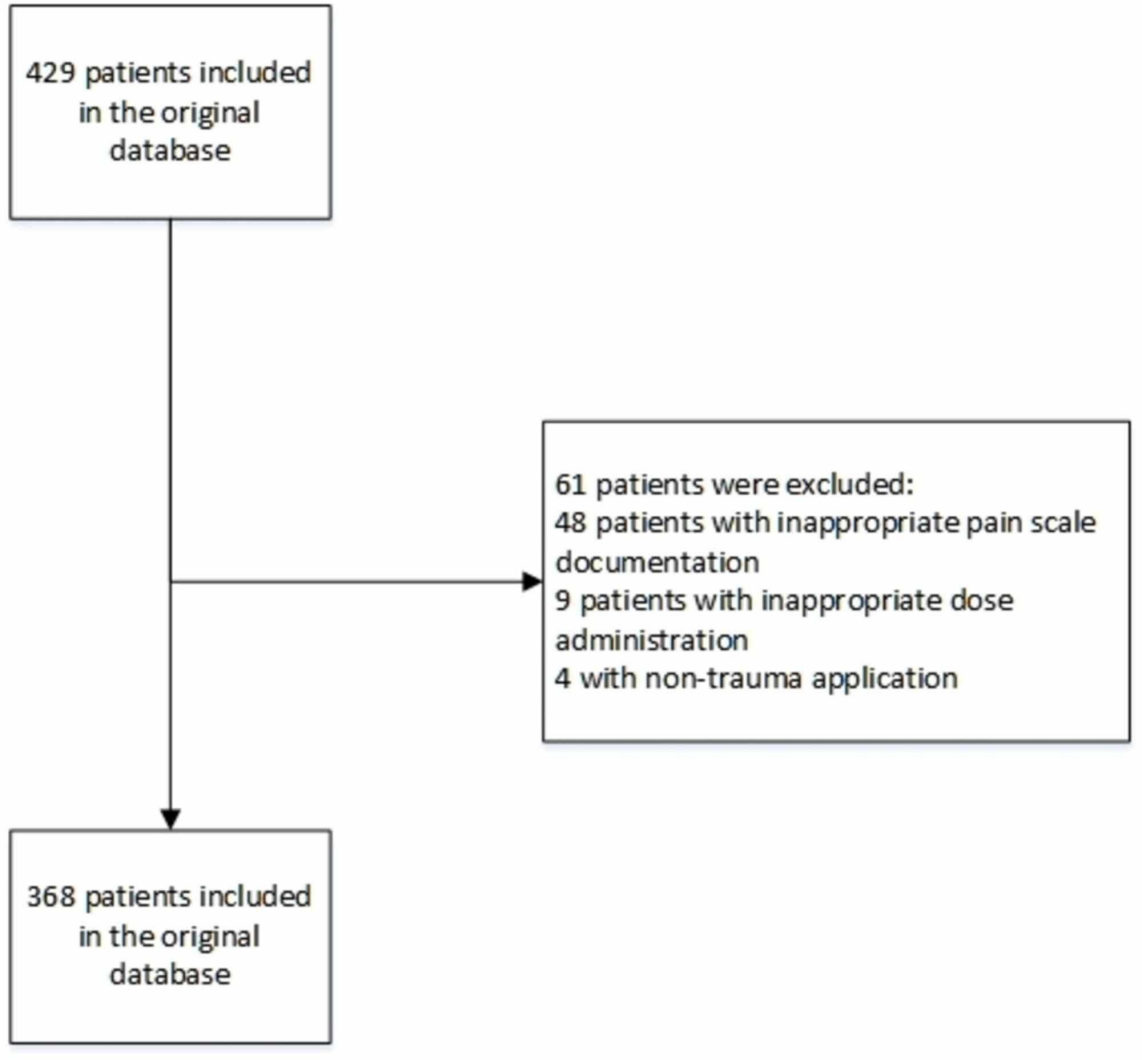
Patients Flow Chart

Among the original 429 patients, 368 patients were included in the final analysis. Figure 1 presents the patient flow chart. The average age was 52.9 ± 23.1 years old, and the average weight was 80.4 ± 22.2 kg. The median transport time from the first administration to arrival at the hospital was 18 minutes (first quartile=12 minutes, third quartile=27 minutes).

The change of pain score and vital signs were compared using a paired T-test. The analysis results were presented in Table 1. There was a statistically significant reduction in the pain score (9.13 ± 1.28 vs 3.7 ± 3.4, delta=5.43 ± 3.38, p<0.0001). Additionally, there was a statistically significant change in SBP (143.42 ± 27.01 vs 145.65 ± 26.26, 2.22 ± 21.1, p=0.0440), pulse (88.06 ± 18 vs 84.64 ± 15.92, delta= -3.42 ± 12.12, p<0.0001), and respiratory rate (19.04 ± 3.59 vs 17.74 ± 3.06, delta=-1.3 ± 2.96, p<0.0001).

**Table 1:** Change of Pain Score and Vital Signs Pre and Post Administration of Ketamine

	Pre	Post	Delta (Post Less Pre)	P-value
Pain Score	9.13 ± 1.28	3.7 ± 3.4	-5.43 ± 3.38	<0.0001
Systolic BP	143.42 ± 27.01	145.65 ± 26.26	2.22 ± 21.1	0.044
Diastolic BP	82.67 ± 17.41	84.42 ± 18.76	1.75 ± 18.29	0.069
Pulse	88.06 ± 18	84.64 ± 15.92	-3.42 ± 12.12	<0.0001
Respiratory Rate	19.04 ± 3.59	17.74 ± 3.06	-1.3 ± 2.96	<0.0001

A total of 58 (15.8%) patients received a second dose of ketamine. A subgroup analysis of change in pain score and vital signs was compared between cohorts who received one vs two doses of ketamine. The analysis results were presented in Table 2. First, among patients who received one dose of ketamine, there was a statistically significant reduction in the pain score (9.05 ± 1.32 vs 3.5 ± 3.35, delta=-5.55 ± 3.35, p<0.0001). Additionally, there was a statistically significant change in pulse (87.64 ± 18.03 vs 84.18 ± 15.91, delta= -3.47 ± 12.08, p<0.0001), and respiratory rate (18.98 ± 3.65 vs 17.73 ± 3.14, delta=-1.24 ± 2.95, p<0.0001). Second, among patients who received two doses of ketamine, there was statistically significant reduction in the pain score (9.59 ± 0.92 vs 4.76 ± 3.5, delta=-4.83 ± 3.53, p<0.0001). Additionally, there was a statistically significant change in respiratory rate (19.37 ± 3.28 vs 17.8 ± 2.64, delta=-1.57 ± 2.99, p=0.0003). Lastly, there was no statistically significant difference in the change of pain score and vital signs between patients who received one vs two doses of ketamine (all p-values>0.05).

**Table 2:** Comparison of Change in Pain Score and Vital Signs Between Ketamine Doses P-value1 was comparing the change of pain score and vital signs among patients who received one dose of ketamine P-value2 was comparing the change of pain score and vital signs among patients who received two doses of ketamine P-value3 was comparing the comparing the change of pain score and vital signs between patients who receive one vs two doses of ketamine.

	One Dose of Ketamine (n=310)				Two Dose of Ketamine (n=58)				
	Pre	Post	Delta (Post Less Pre)	P-value^1^	Pre	Post	Delta (Post Less Pre)	P-value^2^	P-value^3^
Pain Score	9.05 ± 1.32	3.5 ± 3.35	-5.55 ± 3.35	<0.0001	9.59 ± 0.92	4.76 ± 3.5	-4.83 ± 3.53	<0.0001	0.1368
Systolic BP	142.41 ± 26.29	144.47 ± 26.11	2.06 ± 20.71	0.08	148.86 ± 30.24	151.93 ± 26.37	3.07 ± 23.23	0.3186	0.7398
Diastolic BP	81.95 ± 16.36	83.64 ± 18.93	1.7 ± 18.16	0.103	86.56 ± 21.99	88.6 ± 17.36	2.04 ± 19.14	0.4255	0.898
Pulse	87.64 ± 18.03	84.18 ± 15.91	-3.47 ± 12.08	<0.0001	90.3 ± 17.85	87.11 ± 15.89	-3.19 ± 12.41	0.057	0.8756
Respiratory Rate	18.98 ± 3.65	17.73 ± 3.14	-1.24 ± 2.95	<0.0001	19.37 ± 3.28	17.8 ± 2.64	-1.57 ± 2.99	0.0003	0.4548

The complications associated with ketamine were reported in Table 3. Overall, 86.7% (n=319) did not have any complications, and the most prevalent complication was dysphoria (6.3%, n=23). The same pattern of complications apply to patients regardless whether they received one or two doses of ketamine.

**Table 3:** Complications Associated With Administration of Ketamine

	Overall	1 dose	2 doses
Apnea	1 (0.3%)	1 (0.3%)	0 (0%)
Dizziness	11 (3%)	10 (3.2%)	1 (1.7%)
Dysphoria	23 (6.3%)	20 (6.5%)	3 (5.2%)
Euphoria	1 (0.3%)	0 (0%)	1 (1.7%)
Headache	1 (0.3%)	1 (0.3%)	0 (0%)
Hypotension	1 (0.3%)	1 (0.3%)	0 (0%)
Nausea	10 (2.7%)	9 (2.9%)	1 (1.7%)
None	319 (86.7%)	267 (86.1%)	52 (89.7%)
Vomiting	1 (0.3%)	1 (0.3%)	0 (0%)

## Discussion

The current study suggested the effective reduction of acute pain with the use of sub-dissociative doses of ketamine in the prehospital setting. Furthermore, the treatment of pain was not associated with clinically significant adverse effects. While dysphoria was described in a small percentage of patients, hemodynamic instability or respiratory depression were not witnessed. In contrast, opiates, while effective in reducing pain, have a known side effect profile of respiratory depression and hypotension. Similar findings were reported in the literature. One systematic review and meta-analysis, including six trials and 438 patients, suggested that ketamine’s analgesic effects to be similar or superior to placebo or opiates, and while ketamine potentially had increased risk of neurological and psychological adverse effects, the opioid group had a higher risk of major cardiopulmonary events [19].

Other studies have evaluated the use of ketamine in conjunction with opiates to treat acute pain in the emergency department. These studies aim to determine if co-administration reduces the overall use of opiates, and consequentially, the associated adverse effects. The results demonstrate a clinically significant reduction in pain, comparable to opiate alone, with less need for rescue analgesia and decreased incidence of clinically deleterious adverse effects [9].

The opioid epidemic has increased the demand for a less-addictive alternative pharmacologic means of treating acute pain. Several studies demonstrated anti-depressant and anti-addiction properties of ketamine. One study suggested that ketamine may be used to treat addiction by decreasing the incentive-motivational value of reward-related cues [20]. Other studies have shown a prolongation of abstinence from the use of alcohol and heroin in dependent individuals [21]. This suggests that ketamine may be less addicting when compared to opiates, and ketamine may actually aid in preventing addiction to other substances. An article suggested that ketamine’s effect on NMDA receptors on gamma-aminobutyric acid neurons in the thalamic reticular nucleus may lead to increased release of dopamine, thus leading to possible dependence [22]. Death from direct toxicity from ketamine use is rare [23]. In fact, a case report described a case of a patient taking 1-3 g of ketamine daily for multiple years without significant adverse effects. The patient described antidepressant effects, as well as diminished cravings for alcohol [24].

This study has several limitations. First, this study focused on the prehospital setting, and was therefore limited in its capacity to assess whether the use of ketamine may lead to dependence or addiction. However, research has reported the anti-addiction property of ketamine [20-22]. A prospective cohort study with a longer follow-up period is warranted to verify the anti-addiction property of ketamine reported by the research community. Second, prehospital transport time may vary. Longer transportation may increase the total amount of ketamine administered, or demand repeat doses. Conversely, shorter transport time may not have provided sufficient time to establish IV access or administer ketamine as slow intravenous push. Consideration of alternative means of administering ketamine, including intranasal or intramuscular, may potentially circumvent this limitation. Last, severity of traumatic or burn injuries were not provided in the electronic patient care report. Increasing severity of injury may require increased dose, repeat dose, or alternative mechanisms of analgesia for sufficient pain control. One study suggests that Injury Severity Score > 15 required additional doses of analgesia [25]. Furthermore, increased severity of injuries may lead to an increased likelihood of hemodynamic instability following analgesic administration. The current study suggested the positive effect of ketamine administration on systolic blood pressure.

The current research was presented as a podium presentation at the 10th Mediterranean Emergency Medicine Congress in Dubrovnik, Croatia in September 2019. The abstract was published in Western Journal of Emergency Medicine Volume 20 Supplement (5.1). The web address for the published abstract was https://escholarship.org/uc/item/40k6b3fx.

## Conclusions

There is sufficient support for the use of ketamine as a safe alternative to opiates for the treatment of acute pain. Ketamine’s use as an analgesic is well documented in the emergency department, perioperative and inpatient settings. Limited studies in combat demonstrate its efficacy in the prehospital setting. As this study suggests, its use as an analgesic may be expanded to include prehospital administration for civilian populations. Our assessment via the NRS, demonstrated an absolute reduction of pain following the administration of ketamine. Ketamine is safe to administer as slow intravenous push. The trial did not demonstrate severe adverse effects, including hemodynamic instability or respiratory failure. Ketamine appears to be a suitable alternative to opiates for analgesia in the prehospital setting.

Future studies are needed to evaluate for the potential of dependence and abuse, determining the safety and efficacy of ketamine use in the pediatric population, and alternative routes of administration, including intranasal and intramuscular.
